# Identification of Aggregation Pheromone as an Attractant for *Odontothrips loti*, A Serious Thrips Pest on Alfalfa

**DOI:** 10.1007/s10886-024-01532-8

**Published:** 2024-08-12

**Authors:** Xiaowei Li, Jianghui Cheng, Haibin Han, William D.J. Kirk, Matthew O’Brien, Likun Wang, Limin Chen, Haixia Zhang, Zhijun Zhang, Farman Ullah, Nicolas Desneux, Yaobin Lu

**Affiliations:** 1 State Key Laboratory for Managing Biotic and Chemical Threats to Quality and Safety of Agro-products, Key Laboratory of Biotechnology in Plant Protection of MOA of China and Zhejiang Province, Institute of Plant Protection and Microbiology, Zhejiang Academy of Agricultural Sciences, Hangzhou, 310021 China; 2 Institute of Grassland Research, Chinese Academy of Agricultural Sciences, Huhhot, 010010 China; 3 School of Life Sciences, Keele University, Staffordshire, ST5 5BG UK; 4 School of Chemical and Physical Sciences, Keele University, Staffordshire, ST5 5BG UK; 5 Key Laboratory of Pollution Exposure and Health Intervention of Zhejiang Province, Interdisciplinary Research Academy, Zhejiang Shuren University, Hangzhou, 310015 China; 6 Université Côte d’Azur, INRAE, CNRS, UMR ISA, Nice, 06000 France; 7 Institute of Bio-Interaction, Xianghu Laboratory, Hangzhou, 311258 China

**Keywords:** Alfalfa, Thrips, *Odontothrips loti*, Aggregation pheromone, (*R*)-lavandulyl (*R*)-2-methylbutanoate, Attractants, IPM

## Abstract

*Odontothrips loti* (Haliday) (Thysanoptera: Thripidae) is one of the most serious pests on alfalfa, causing direct damage by feeding and indirect damage by transmitting plant viruses. This damage causes significant loss in alfalfa production. Semiochemicals offer opportunities to develop new approaches to thrips management. In this study, behavioral responses of female and male adults of *O. loti* to headspace volatiles from live female and male conspecifics were tested in a Y-tube olfactometer. The results showed that both male and female adults of *O. loti* were attracted to the odors released by conspecific males but not those released by females. Headspace volatiles released by female and male adults were collected using headspace solid-phase microextraction (HS-SPME). The active compound in the volatiles was identified by gas chromatography-mass spectrometry (GC-MS). The analysis showed that there was one major compound, (*R*)-lavandulyl (*R*)-2-methylbutanoate. The attractive activity of the synthetic aggregation pheromone compound was tested under laboratory and field conditions. In an olfactometer, both male and female adults showed significant preference for synthetic (*R*)-lavandulyl (*R*)-2-methylbutanoate at certain doses. Lures with synthetic (*R*)-lavandulyl (*R*)-2-methylbutanoate significantly increased the trap catches of sticky white traps at doses of 40–80 µg in the field. This study confirmed the production of aggregation pheromone by *O. loti* male adults and identified its active compound as (*R*)-lavandulyl (*R*)-2-methylbutanoate, providing a basis for population monitoring and mass trapping of this pest.

**Supplementary Information:**

The online version contains supplementary material available at 10.1007/s10886-024-01532-8.

## Introduction

Alfalfa (*Medicago sativa* L.), a forage legume herb rich in protein, dietary fiber, vitamins, and minerals, is among the most important perennial forage crops in animal husbandry, and has been widely planted worldwide (Ma et al. 2022; Radovic et al. 2009). In China, due to the rapid development of animal husbandry and the policy of returning food crop farmland to forest and grassland, the cultivation area of alfalfa has continued to expand (McNeill et al. 2021; Wang et al. 2023; Zhang et al. 2023). In the major alfalfa cultivation areas, such as Inner Mongolia, Gansu, and Ningxia, *Odontothrips loti* (Haliday) is one of the most serious pests on alfalfa (McNeill et al. 2021) and has also been reported to cause serious damage to other leguminous plants in Europe and Africa (Laamari 2013; Virteiu et al. 2016). This pest causes direct damage to alfalfa by feeding on leaves, stems, and flowers and indirect damage by transmitting plant viruses (e.g., *Alfalfa mosaic virus*) (Li et al. 2021, 2022). This causes more than 35% decrease in plant height and leaf area, as well as more than 20% loss in alfalfa production (Li et al. 2022).

Currently, the control of thrips pests in alfalfa fields is heavily reliant on chemical control. However, the intensive use of chemical insecticides has resulted in insecticide resistance, the accumulation of pesticide residues, and environmental pollution (Pan et al. 2023). Consequently, traditional use of insecticides is no longer the best option, and more focus is being placed on the development of alternative strategies within the context of integrated pest management (IPM)(McNeill et al. 2021).

Semiochemicals, such as thrips-produced pheromones and plant-released semiochemicals, offer opportunities to develop new approaches to thrips pest management (Kirk et al. 2021). Aggregation pheromones, which are produced by adult male thrips and attract both female and male adults, have been studied intensively in thrips. The active compounds have been identified in five thrips species, including *Frankliniella occidentalis* (Hamilton et al. 2005), *Frankliniella intonsa* (Zhang et al. 2011), *Thrips palmi* (Akella et al. 2014), *Megalurothrips sjostedti* (Niassy et al. 2019) and *Megalurothrips* *usitatus* (Li et al. 2019b; Liu et al. 2020). There are many ways in which aggregation pheromones could potentially be used to manage thrips pests, including monitoring, mass trapping, mating disruption, activators for insecticides and biologicals, push-pull, lure and infect, etc. (Kirk 2017; Kirk et al. 2021). Current applications are mainly used for monitoring and mass trapping. For instance, the major compound of the aggregation pheromone of *F. occidentalis* has been commercially developed into lures (Thripline from Bioline AgroSciences and ThriPher from Biobest), which increase thrips captures with sticky traps, leading to earlier and more accurate monitoring, and reduction of thrips damage in the field (Sampson and Kirk 2013).

The identification of thrips-released aggregation pheromones and development of pheromone-based attractants can contribute to the development of novel and ecologically friendly control methods for thrips. In this study, we obtained behavioral evidence for aggregation pheromone produced by male *O. loti* based on Y-tube olfactometer bioassays, and identified the active compound of *O. loti* aggregation pheromone using headspace-solid phase microextraction (HS-SPME) and gas chromatography-mass spectrometry (GC-MS). The attraction of the synthetic compound was confirmed under laboratory and field conditions. These results provided the basis for future development of aggregation pheromone attractants for this important pest.

## Methods and Materials

### Thrips Populations

The population of *O. loti* was collected from alfalfa in the field at the Experimental Station of the Institute of Grassland Research, Chinese Academy of Agricultural Sciences, Huhhot, China (111°47’12″E, 40° 34’ 52″ N). Cultures of this thrips species were reared continuously on alfalfa plants in cages in climate rooms at 25 ± 1 °C under a LD 16:8 h photocycle and 65–75% relative humidity.

### Olfactometer Bioassays

The responses of female and male adults of *O. loti* to female-produced or male-produced volatiles were tested in a Y-tube olfactometer in a dark room at 25 ± 2 °C, RH 60 ± 5%. Light was provided by LED light immediately above the Y-tube. The glass Y-tube olfactometer consisted of a base tube (60 mm long, 10 mm in diameter), and two arms (60 mm long, 10 mm in diameter) at an angle of 90°. Air was pumped into the apparatus by an electromagnetic air pump (ACO serial, Sunsun Group Co., LTD, China) and filtered through activated charcoal and distilled water, and split into two air streams at a flow rate of 60 mL/s for each arm, each of which was fed through a glass flask and into one arm of the olfactometer. The two glass flasks (60 mL) provided odor sources. Newly emerged female and male adult thrips used for olfactometer bioassays were collected from the cages. Fifty males or females were transferred into the treatment flask as the test odor source. An empty flask was the control odor source. The two flasks were illuminated by cold LED light at 10 000 lx. Teflon tubes were used to connect the components of the olfactometer apparatus.

Test thrips were transferred individually to the base tube of the Y-tube. The choice of each thrips was recorded when it crossed a half-length of either arm within 5 min. ‘No choice’ was recorded if the test thrips did not cross a half-length of either arm within 5 min. Thrips that made ‘No choice’ were not included in the statistical analysis. After five thrips were tested, odor sources entering the arms of the Y-tube were swapped to avoid potential bias in the apparatus. The experiment included 100 thrips for each odor comparison. The apparatus was cleaned before each different odor by rinsing with pure ethanol and dried in an oven (120 °C).

### Collection and Analysis of Volatile Compounds from Female and Male Adults of *O. loti*

The volatile compounds from female and male adults were collected by headspace-solid phase microextraction (HS-SPME). A 100-µm polydimethylsiloxane SPME fiber assembly (Supelco, Bellefonte, Pennsylvania) was preconditioned by heating at 250 °C for 1 h. Forty newly emerged females or males were transferred into a 1.5 mL glass container, and illuminated by cold light at 10 000 lx. Headspace volatiles were collected on the SPME fiber at 25 ± 1 °C for 4 h.

The volatile compounds collected by HS-SPME were analyzed by gas chromatography-mass spectrometry (GC-MS) using a Shimadzu GC-MS-QP2010 plus (Shimadzu, Japan), equipped with a Rxi-5ms column (30 m × 0.25 mm i.d., 0.25 μm film thickness, Restek Corp., Bellefonte, Pennsylvania), and a chiral CycloSil-B column (30 m, 0.25 mm i.d., 0.25 μm film thickness, Agilent Technologies). The SPME sample was injected into the inlet port (250 °C) in the splitless mode and desorbed for 5 min before the fiber assembly was withdrawn. The carrier gas was helium (1 ml/min). The temperature program of the GC was as follows: initial temperature of 40 °C for 2 min, then increased at 10 °C/min to 150 °C (hold for 0 min), at 1 °C/min to 180 °C (hold for 0 min), and finally at 10 °C/min to 200 °C (hold for 1 min). The MS detector was operated in electron impact mode (70 eV) and mass spectra were recorded from 30 to 350 amu. The volatile compounds were identified by comparing their mass spectra with the NIST 20s mass spectra library. Based on the similarity search results and mass spectra, the identity of the target compound was further confirmed by comparing the retention time of 12 possible synthetic racemic monoterpene C5 esters (neryl valerate, neryl 2-methylbutanoate, neryl 3-methylbutanoate, geranyl valerate, geranyl 2-methylbutanoate, geranyl 3-methylbutanoate, lavandulyl valerate, lavandulyl 2-methylbutanoate, lavandulyl 3-methylbutanoate, linalyl valerate, linalyl 2-methylbutanoate, and linalyl 3-methylbutanoate, ordered from Bidepharm, Shanghai, China) and co-injection on the GC-MS equipped with a chiral CycloSil-B column. For co-injection, HS-SPME fiber was used to collect volatiles from male adults for 2 h, then the HS-SPME fiber was immediately transferred to another glass container, containing a piece of filter paper with 10 µL of 10 ng/µL synthetic monoterpene C5 esters. The synthetic volatiles were collected for a further 1 h. The selected temperature program of the GC separated the compounds but not the enantiomers, and was as follows: initial temperature of 55 °C for 2 min, then increased at 10 °C/min to 150 °C (hold for 1 min), and finally at 1 °C/min to 160 °C.

### Chiral Chromatography of the Identified Compound

The identified compound from male volatiles, lavandulyl 2-methylbutanoate, has four stereoisomers. The absolute configuration of natural compound was determined by hydrolysis and chiral column separation on GC-MS. Firstly, the absolute configuration of the alcohol was determined by the modified hydrolysis method reported previously (Ho et al. 2009; Zhang et al. 2004). Male-produced natural compound (Fig S1A) was extracted by immersing 300 male thrips in 500 µl hexane for at least 24 h. Extracts were filtered and concentrated under a stream of nitrogen. 300 µl ethanol and two drops of 2 M NaOH were added. The reaction mixture was kept in a water bath at 35℃ for 1 h. Then 0.5 ml water was added, and the aqueous phase was extracted twice with 0.5 ml hexane. The combined organic layers containing hydrolyzed alcohol were concentrated under nitrogen, and analyzed together with (*R*)-lavandulol (95%, Bidepharm, Shanghai, China) and (*S*)-lavandulol (95%, Bidepharm, Shanghai, China) on a GC-MS equipped with a chiral CycloSil-B column. The temperature program of the GC, which was adjusted to separate the enantiomers, was as follows: initial temperature of 55 °C for 1 min, then increased at 5 °C/min to 160 °C (held for 1 min), and finally at 10 °C/min to 200 °C.

Based on hydrolysis results, the ester showed (*R*)-configuration of the alcohol part. Then (*R*)-lavandulyl (*R*)-2-methylbutanoate and (*R*)-lavandulyl (*S*)-2-methylbutanoate were synthesized from esterification of (*R*)-lavandulol with (*R*)-2-methylbutanoate acid (95%, Bidepharm, Shanghai, China) and (*S*)-2-methylbutanoate acid (98%, Bidepharm, Shanghai, China), respectively, according to the method reported before (Akella et al. 2014; Hamilton et al. 2005). Chiral GC showed the two synthesized esters had an enantiomeric excess of over 98% and a purity of over 95%. The natural compound from males, and the two synthesized esters were analyzed by GC-MS equipped with a chiral CycloSil-B column. The temperature program of the GC, adjusted to separate these enantiomers, was as follows: initial temperature of 100 °C for 1 min, then increased at 10 °C/min to 170 °C, and then at 1 °C/min to 180 °C.

### The Responses of *O. loti* to Synthetic Compound in Olfactometer Bioassays

The responses of female and male adults of *O. loti* to synthetic compound (*R*)-lavandulyl (*R*)-2-methylbutanoate were evaluated using a Y-tube olfactometer as described above. (*R*)-Lavandulyl (*R*)-2-methylbutanoate was prepared in hexane at four concentrations (0.01, 0.1, 1, 10 ng/µL), and 10 µL of the test solution was applied to a piece of filter paper (1 cm × 1 cm), which was placed in the 60 mL glass flask. The resultant doses on the filter papers were 0.1, 1, 10 and 100 ng. Filter paper with 10 µL hexane in a glass flask was used as control. Olfactometer bioassays were conducted as detailed above. The filter papers with the test solution and hexane were replaced every 20 min. The experiment included 100 thrips for each odor comparison.

### Field Trial

Field trials were conducted in September 2023 in the alfalfa field at the Experimental Station of the Institute of Grassland Research, Chinese Academy of Agricultural Sciences, Huhhot, China. Since the field attractive doses of aggregation pheromone in other thrips species were 30 ~ 90 µg (Akella et al. 2014; Hamilton et al. 2005; Liu et al. 2020), three doses of synthetic compound (*R*)-lavandulyl (*R*)-2-methylbutanoate (40, 60 and 80 µg) in hexane and solvent control (30 µL hexane) were loaded into rubber septa (1 cm diameter × 1.5 cm long, pre-cleaned with hexane). The rubber septa were stuck to the middle of rectangular white sticky traps (20 cm × 25 cm) hung 10 cm above crop height. White has been reported to be the most attractive color of trap for *O. loti* (Qiao et al. 2023). In the field, 4 treatments were randomly assigned to a block in the same row, with 8 traps for each treatment. The traps were 2 m apart from each other. The numbers of females and males on each trap were recorded after 24 h.

### Statistical Analysis

All data analyses were performed in SPSS Version 22. Olfactometer data were analyzed using the chi-square test to determine significant deviation (*P* < 0.05) from an expected 1:1 response. The distributions of count data from field trials at different doses were checked using nonparametric Kolmogorov-Smirnov tests (*P* < 0.05), and analyzed using Generalized Linear Models with Poisson distributions followed by LSD multiple range tests (*P* < 0.05).

## Results

### Behavioral Responses of Adult *O. loti* to Female-Produced or Male-Produced Volatiles

**Fig. 1:**
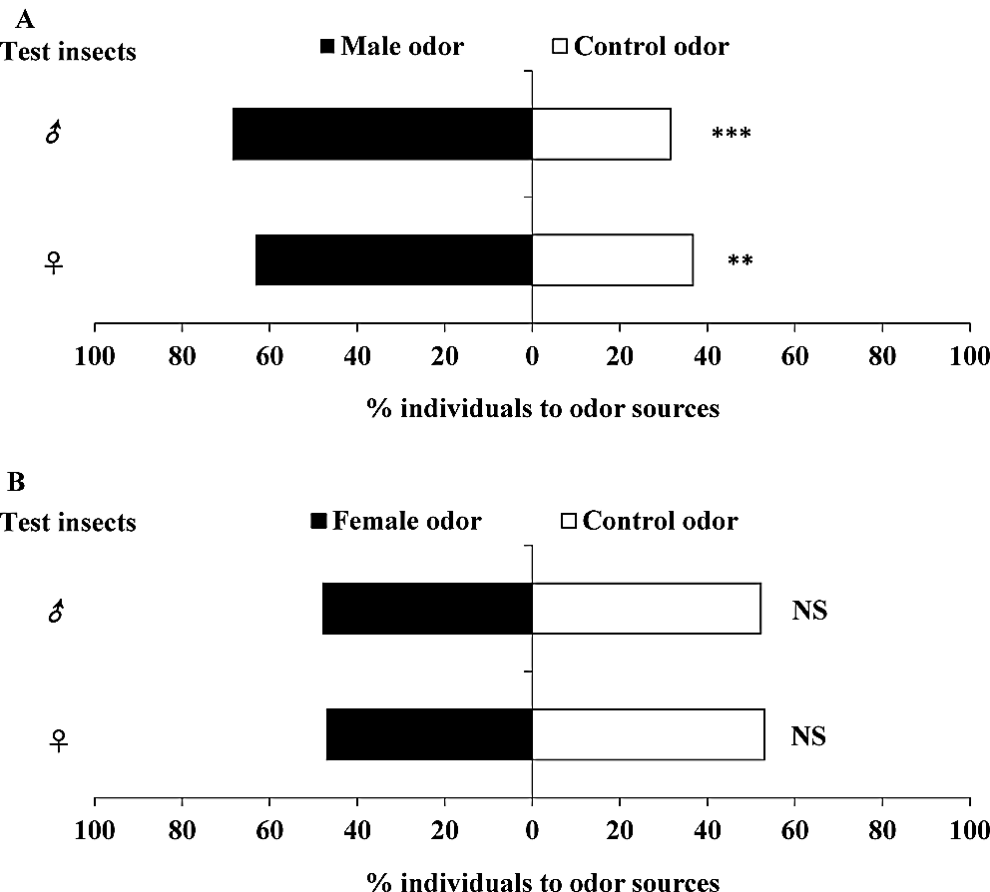
Responses of *Odontothrips loti* female and male adults to male (**A**) and female (**B**) produced volatiles. ♀: female adults; ♂: male adults; Asterisks indicate significant differences within a choice test (*P < 0.05; **P < 0.01; ***P < 0.001); NS indicates no significant difference

Females and males of *O. loti* were significantly attracted by the volatiles from male adults relative to clean air (Fig. 1A; female: *χ*^2^ = 6.760, *df* = 1, *P* = 0.009; male: *χ*^2^ = 12.960, *df* = 1, *P* < 0.0001), with 63.3% of females and 68.4% of males choosing male-produced volatiles compared with control. In contrast, females and males of *O. loti* showed no preference for female-produced volatiles (Fig. 1B; female: *χ*^2^ = 0.360, *df* = 1, *P* = 0.549; male: *χ*^2^ = 0.160, *df* = 1, *P* = 0.689). These results indicated that male adults of *O. loti* produce aggregation pheromone that attracts conspecifics of both sexes.

### Identification of Male-Produced Compounds by GC-MS Analysis

**Fig. 2:**
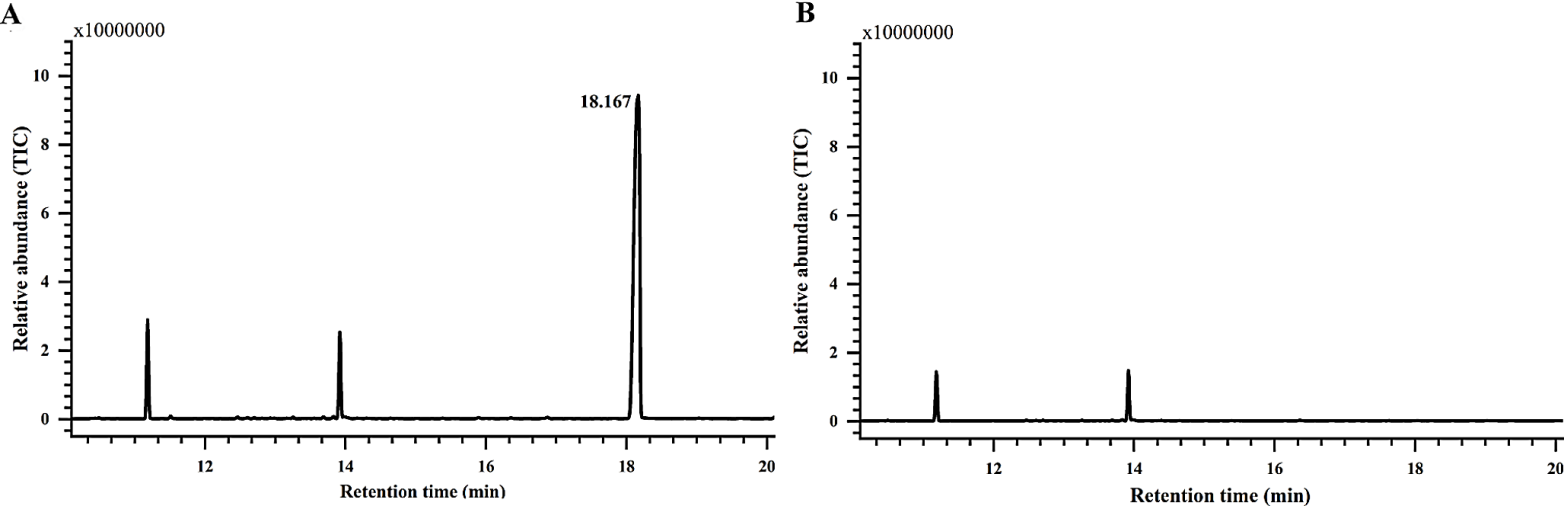
Chromatograms of the headspace volatiles from adult males (**A**) and females (**B**) of *Odontothrips loti* on a Rxi-5ms column

**Fig. 3:**
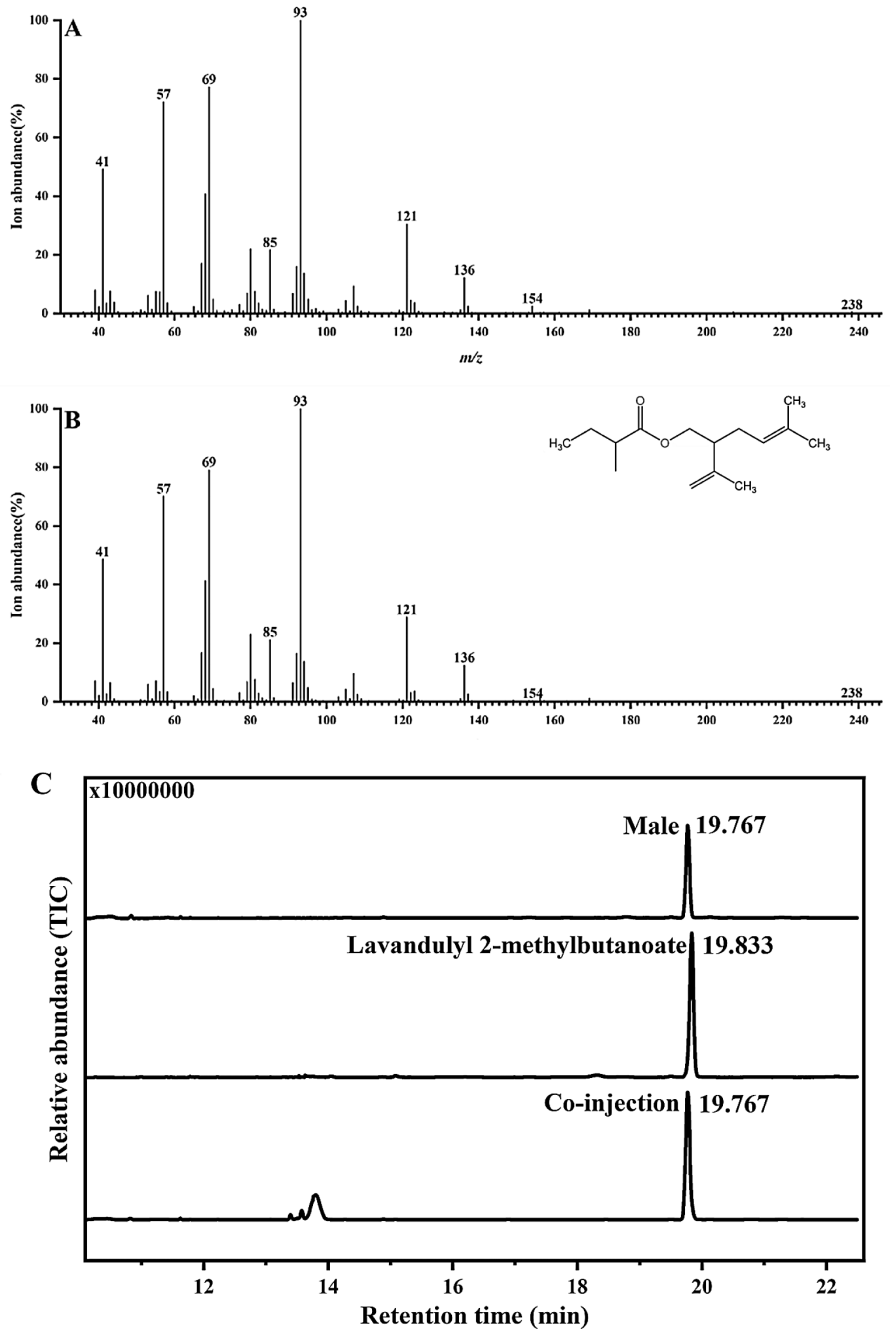
Identification of male-produced aggregation pheromone compound by GC-MS analysis. **A** and **B**: EI mass spectra of the *Odontothrips loti* male-produced compound (**A**) and synthetic lavandulyl 2-methylbutanoate (**B**). **C**: Chromatograms of the headspace volatiles from adult males, lavandulyl 2-methylbutanoate, and co-injection of male headspace volatiles and lavandulyl 2-methylbutanoate

**Fig. 4:**
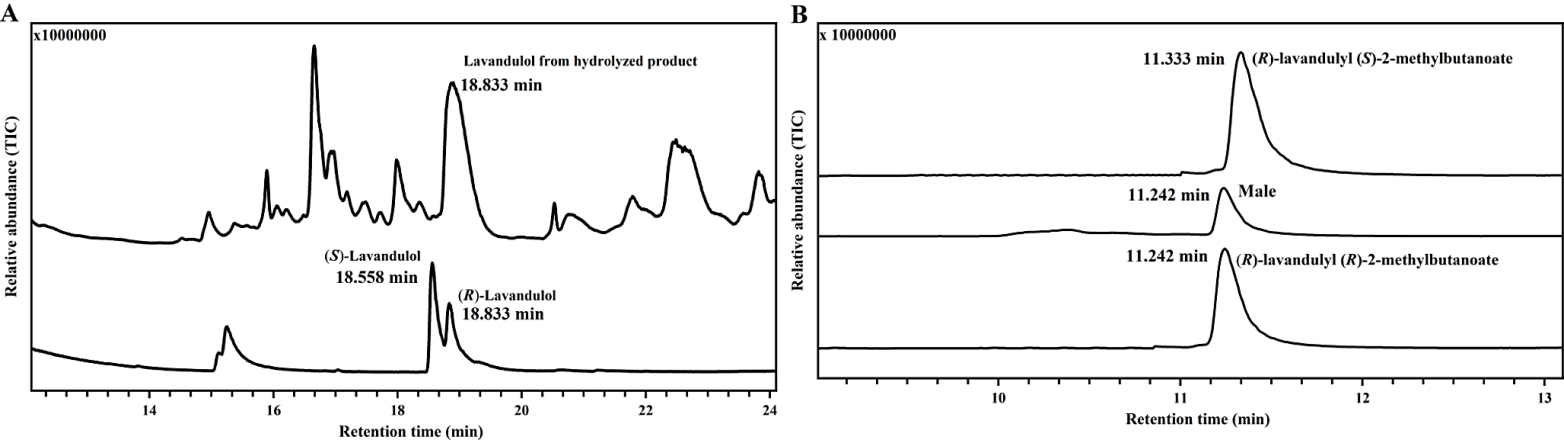
Chiral chromatography of the male-produced aggregation pheromone. **A**: Comparison of chromatograms of hydrolyzed alcohol, (*R*)-lavandulol and (*S*)-lavandulol. **B**: Comparison of chromatograms of male-produced natural compound, (*R*)-lavandulyl (*R*)-2-methylbutanoate and (*R*)-lavandulyl (*S*)-2-methylbutanoate

A comparison of total ion chromatograms from GC-MS analyses showed that there was one distinct peak (retention time 18.167 min) present in the volatiles of adult males (Fig. 2A) but absent in the volatiles of adult females (Fig. 2B). Comparative analysis of the results from the non-polar column and the polar column (Fig. S1A and Fig. 4B) confirmed that only one component was present. The presence of a trace ion at *m*/*z* 154 (0.2%), but strong ions at *m*/*z* 136 (12%), 121 (29%), 93 (100%), 69 (79%) and 41 (49%) in mass spectra suggested that there was a monoterpene substructure (Fig. 3A). An ion at *m*/*z* 238 (0.4%) suggested a molecular weight of 238 (Fig. 3A). The presence of the ions at *m*/*z* 85 (21%) and 57 (70%) suggested the loss of C_4_H_9_CO + and C_4_H_9_ + fragments respectively derived from a saturated 5-carbon acid moiety (Fig. 3A). These results suggested that the compound was an ester of monoterpene alcohol C_10_H_18_O and saturated 5-carbon acid C_5_H_10_O_2_.

The comparison of the retention time of male-produced compound and 12 possible synthetic monoterpene C5 esters on a chiral CycloSil-B column revealed that the male-produced compound had identical retention time to lavandulyl 2-methylbutanoate (Fig. 3C). Co-injection of male headspace volatiles and lavandulyl 2-methylbutanoate gave an enhanced single peak at 19.767 min, suggesting the male-produced compound was lavandulyl 2-methylbutanoate (Fig. 3C). In addition, the mass spectra of natural compound and lavandulyl 2-methylbutanoate were superimposable (Fig. 3B). The small peaks at around 14 min (Fig. 3C) were considered to be contaminants.

**Fig. 5:**
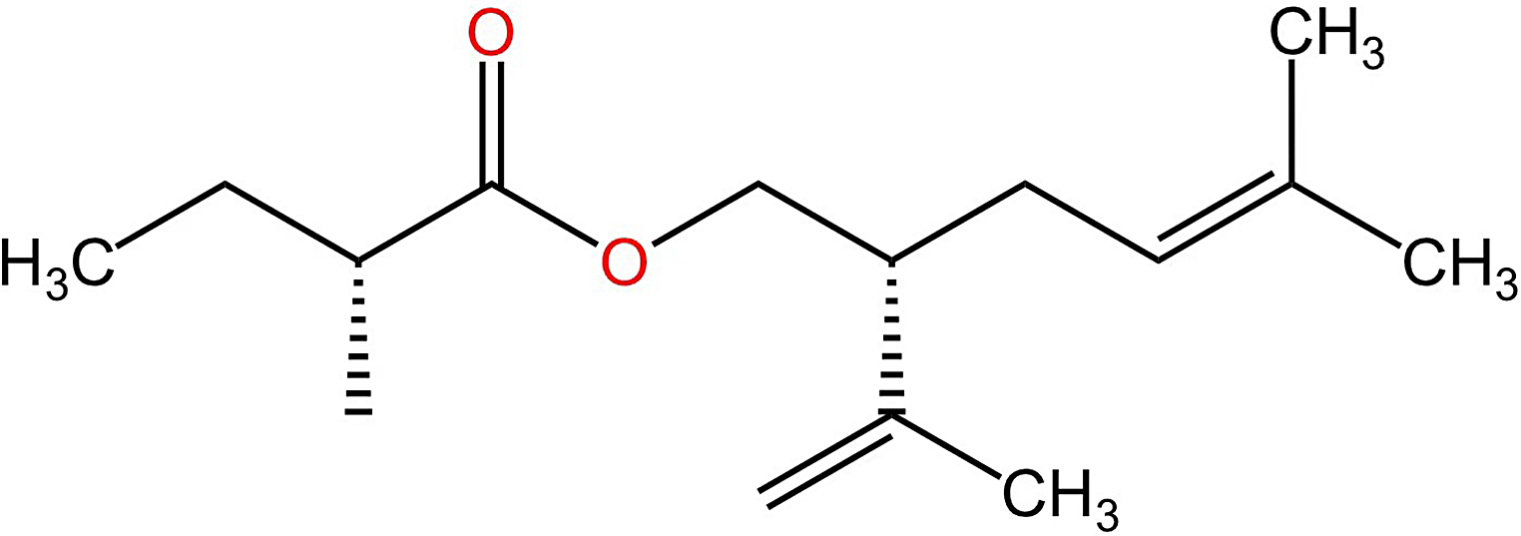
Structure of the identified aggregation pheromone compound of male *Odontothrips loti*, (*R*)-lavandulyl (*R*)-2-methylbutanoate

Lavandulyl 2-methylbutanoate has four different stereoisomers. However, we could not separate the four stereoisomers, despite trying several chiral columns and various temperature programs, whereas (*R*)- lavandulol and (*S*)- lavandulol could be separated easily on a chiral column. Consequently, the absolute configuration of naturally produced compound was determined by hydrolysis and chiral column separation on GC-MS. On a chiral CycloSil-B column, (*R*)-lavandulol and (*S*)-lavandulol gave two peaks at 18.833 min and 18.558 min, respectively. The hydrolyzed alcohol from hexane extracts of males (Fig. S1A) gave a peak at 18.833 min, suggesting the naturally produced ester showed (*R*)-configuration at the alcohol (Fig. 4A). Then the natural compound from males was compared on a chiral column with two synthesized esters: (*R*)-lavandulyl (*R*)-2-methylbutanoate and (*R*)-lavandulyl (*S*)-2-methylbutanoate. Male-produced natural compound had the same retention time as (*R*)-lavandulyl (*R*)-2-methylbutanoate (11.242 min), but separated from (*R*)-lavandulyl (*S*)-2-methylbutanoate (11.333 min) (Fig. 4B). Consequently, the male-produced natural compound was confirmed as (*R*)-lavandulyl (*R*)-2-methylbutanoate (Fig. 5).

### The Responses of *O. loti* to Synthetic Compound in Olfactometer Bioassays

**Fig. 6:**
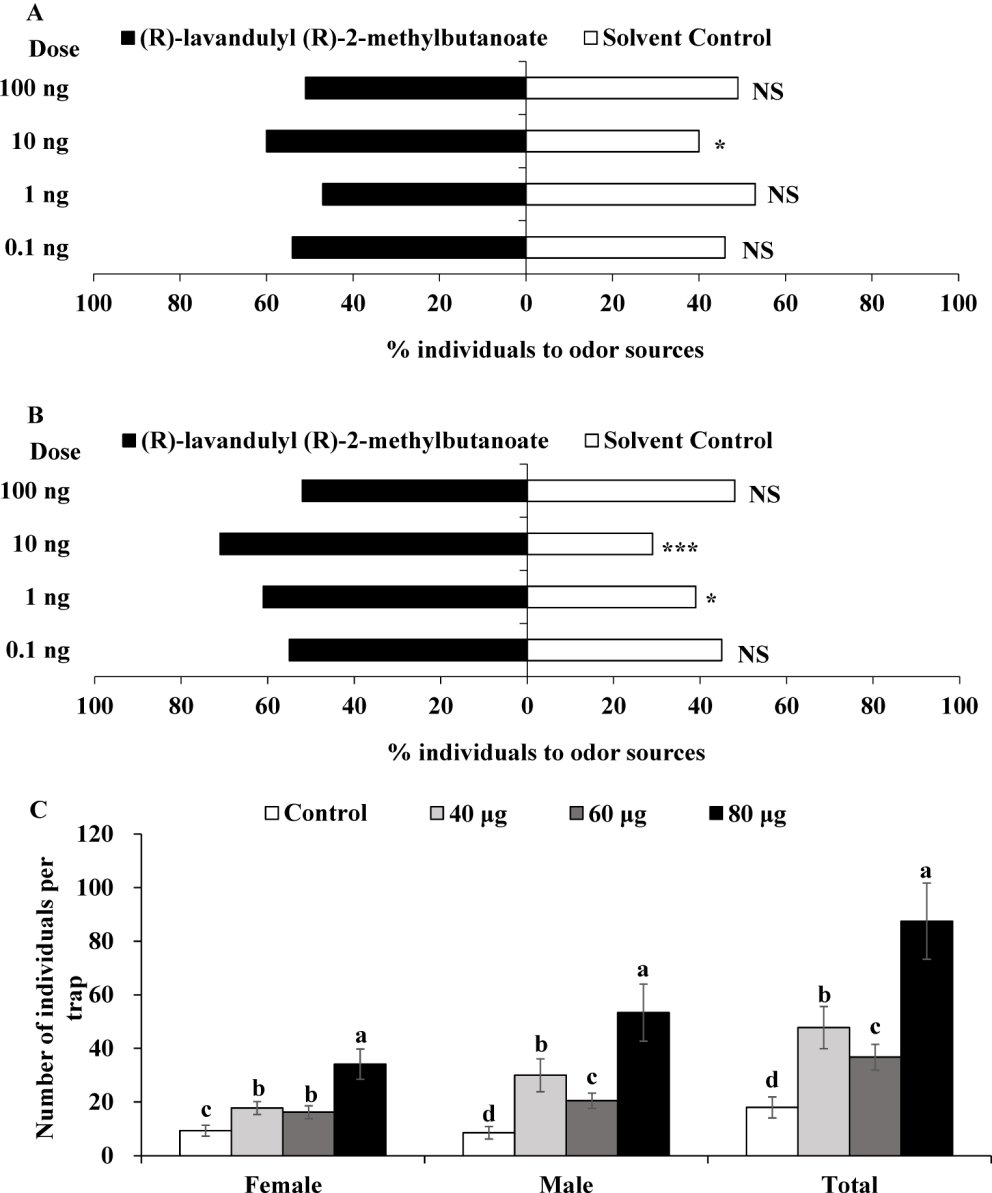
The attractive activity of synthetic compound (*R*)-lavandulyl (*R*)-2-methylbutanoate to *Odontothrips loti* in the laboratory and field. **A** and **B**: Responses of *O. loti* female (**A**) and male (**B**) adults to synthetic compound (*R*)-lavandulyl (*R*)-2-methylbutanoate at four doses; asterisks indicate significant differences within a choice test (*P < 0.05; **P < 0.01; ***P < 0.001), NS indicates no significant difference. **C**: Average (± SE) number of females, males, and total *Odontothrips loti* caught by sticky traps with synthetic (*R*)-lavandulyl (*R*)-2-methylbutanoate lures at three doses and a control (0 µg); different letters above columns indicate significant differences (*P* < 0.05)

Y-tube olfactometer bioassays showed that both female and male adults of *O. loti* were significantly attracted to synthetic (*R*)-lavandulyl (*R*)-2-methylbutanoate. Specifically, (*R*)-lavandulyl (*R*)-2-methylbutanoate showed significant attractive effects on *O. loti* females at a dose of 10 ng (Fig. 6A; *χ*^2^ = 4.00, *df* = 1, *P* = 0.046), and significant attractive effects on *O. loti* males at doses of 1 and 10 ng (Fig. 6B; 1 ng: *χ*^2^ = 4.84, *df* = 1, *P* = 0.028; 10 ng: *χ*^2^ = 17.64, *df* = 1, *P* < 0.0001).

### Field Trials

Compared with control traps, traps with 40, 60, or 80 µg of synthetic (*R*)-lavandulyl (*R*)-2-methylbutanoate caught significantly more females, males, and total number of *O. loti* (Fig. 6C; female: *χ*^2^ = 115.76, *df* = 3, *P* < 0.0001; male: *χ*^2^ = 309.57, *df* = 3, *P* < 0.0001; total: *χ*^2^ = 408.29, *df* = 3, *P* < 0.0001). Traps with 80 µg synthetic (*R*)-lavandulyl (*R*)-2-methylbutanoate caught the most *O. loti*, catching 3.6, 6.2, and 4.9 times more than the control for females, males, and the total number of *O. loti*, respectively.

## Discussion

Aggregation behavior has been described in several thrips species and appears to be mediated by male-produced aggregation pheromones (Kirk et al. 2021). Male adults of *O. loti* produced volatiles that attracted both female and male adult conspecifics, indicating the presence of a male-produced aggregation pheromone, as reported in other thrips species, i.e. *F. occidentalis*, *F. intonsa*, *T. palmi*, *M. sjostedti*, *M. usitatus*, and *Pezothrips kellyanus* (Kirk et al. 2021; Webster et al. 2006). Characterization of the headspace volatiles of *O. loti* males revealed the presence of only one compound not present in the females, (*R*)-lavandulyl (*R*)-2-methylbutanoate, which has also been reported to be the minor compound of female-produced sex pheromone in Madeira mealybug, *Phenacoccus madeirensis* (Ho et al. 2009). A single compound was also reported in the aggregation pheromone of *T. palmi* (Akella et al. 2014) and *M.* *usitatus* (Li et al. 2019b; Liu et al. 2020), whereas two compounds were reported in *F. occidentalis* (Hamilton et al. 2005), *F.intonsa* (Zhang et al. 2011) and *M*. *sjostedti* (Niassy et al. 2019). (*R*)-lavandulyl (*R*)-2-methylbutanoate is an ester of a monoterpene alcohol and a five-carbon acid. This typical structure has also been reported in most of the identified aggregation pheromone compounds in thrips, i.e. (*R*)-lavandulyl 3-methyl-3-butenoate in *T. palmi* (Akella et al. 2014), (*R*)-lavandulyl 3-methylbutanoate in *M. sjostedti* (Niassy et al. 2019), and neryl (*S*)-2-methylbutanoate in *F. occidentalis* and *F. intonsa* (Hamilton et al. 2005; Zhang et al. 2011). There was one exception in *M. usitatus*, where the only compound, (2*E*,6*E*)-farnesyl acetate, is an ester of a sesquiterpene alcohol and a two-carbon acid (Li et al. 2019b; Liu et al. 2020). The minor compounds, (*R*)-lavandulol in *M. sjostedti*, and (*R*)-lavandulyl acetate in *F. occidentalis* and *F. intonsa*, are both related to the (*R*)-lavandulol structure (Hamilton et al. 2005; Zhang et al. 2011). Comparison of the active compounds of thrips species from different genera suggest that the aggregation pheromone biosynthesis pathway might be highly conserved in the family Thripidae, and that the lavandulol structure plays an important role.

This is the first record of (*R*)-lavandulyl (*R*)-2-methylbutanoate in any species of thrips and the first record of an aggregation pheromone in the genus *Odontothrips*. Thrips aggregation pheromones are likely to be produced by the sternal glands underlying the abdominal sternal pore plates (Kirk et al. 2021), which are found in males of many species of Thripidae (Mound 2009). However, sternal pore plates are reported to be absent in the genus *Odontothrips* (Mound 2009; Pitkin 1972), which would suggest that the aggregation pheromone is produced elsewhere, but closer inspection has shown that there is a single, small basal-central pore plate on each of sternites IV-VII in *O. loti*, which leaves open the possibility that the pheromone is produced by sternal glands.

Our results showed that synthetic (*R*)-lavandulyl (*R*)-2-methylbutanoate was attractive to female and male *O. loti*, both in the laboratory and in the field. In the Y-tube olfactometer bioassays, a narrow range of active doses (10 ng for females, and 1 ~ 10 ng for males) was recorded, which has also been found with some plant volatiles for *F. occidentalis*, i.e. eugenol and benzaldehyde (Koschier et al. 2000), suggesting that the concentration of semiochemicals is critical for behavioral responses of thrips pest species (Koschier and Sedy 2003). In the field trials, all three test doses of synthetic (*R*)-lavandulyl (*R*)-2-methylbutanoate were strongly attractive to *O*. *loti*, with the dose of 80 µg the most attractive. The attractive doses in *O. loti* were similar to those in *M. usitatus*, but higher than those in *F. occidentalis*, *F. intonsa* and *T. palmi* (Akella et al. 2014; Hamilton et al. 2005; Li et al. 2019a). In addition, the effectiveness of doses above 80 µg needs to be investigated further. Thrips did not respond to higher doses in the olfactometer, but were caught by higher doses on traps. However, a direct comparison of these situations is not useful because it compares a walking response to pheromone in a confined space with a flying/landing response to pheromone plus visual stimulus in the open, which is likely to be very different. The actual concentrations for the response are not known in either situation and in the field would vary greatly with time and distance, so cannot be compared.

Aggregation pheromones of thrips can play an important role in reproductive isolation of conspecific species (Li et al. 2019a). It has been reported that aggregation pheromone of one thrips species had no effect on the capture of other thrips species (Akella et al. 2014; Broughton and Harrison 2012). In our field trials, other thrips species, e.g., *F. intonsa*, were also present on the traps, but there were too few individuals for statistical analysis of the effect of the pheromone.

Since aggregation pheromones attract both sexes, they have greater potential for mass trapping than sex pheromones, which attract only one sex. Aggregation pheromones are used as lures attached to or impregnated in colored sticky traps to increase trap catches (Broughton et al. 2015; Kirk et al. 2021). Pheromone lures with sticky traps are applicable for monitoring and mass trapping of thrips pests in fruit, flower, and vegetable production (Broughton et al. 2015; Broughton and Harrison 2012; Covaci et al. 2012). Mass trapping with pheromone lures effectively reduced the thrips damage and was cost-effective in high-value crops in semi-protected or protected fields (Sampson and Kirk 2013). Our results showed that lures with synthetic aggregation pheromone of *O. loti* increased trap catch by a factor of 4.9 at a dose of 80 µg on white traps, which were already visually highly attractive. Consequently, aggregation pheromone lures with white sticky traps could be effective tools for *O. loti* monitoring and mass trapping. However, since alfalfa is planted in large open fields, further studies are needed to evaluate whether this method could reduce thrips damage and whether it is economically viable.

In conclusion, this study provided the behavioral evidence for aggregation pheromone produced by male *O. loti*, which was identified as a single compound, (*R*)-lavandulyl (*R*)-2-methylbutanoate. This compound was highly attractive under both laboratory and field conditions. These results provide the basis for the development of aggregation pheromone attractants for this important pest.

## Electronic supplementary material

Below is the link to the electronic supplementary material.

Supplementary Material 1

## Data Availability

No datasets were generated or analysed during the current study.
