# Supplementary figures and images for: Identification of Aggregation Pheromone as an Attractant for Odontothrips loti, A Serious Thrips Pest on Alfalfa
Source: J Chem Ecol. 2024 Aug 12;50(12):894–903. doi: 10.1007/s10886-024-01532-8 (PMC11717801; doi:10.1007/s10886-024-01532-8)

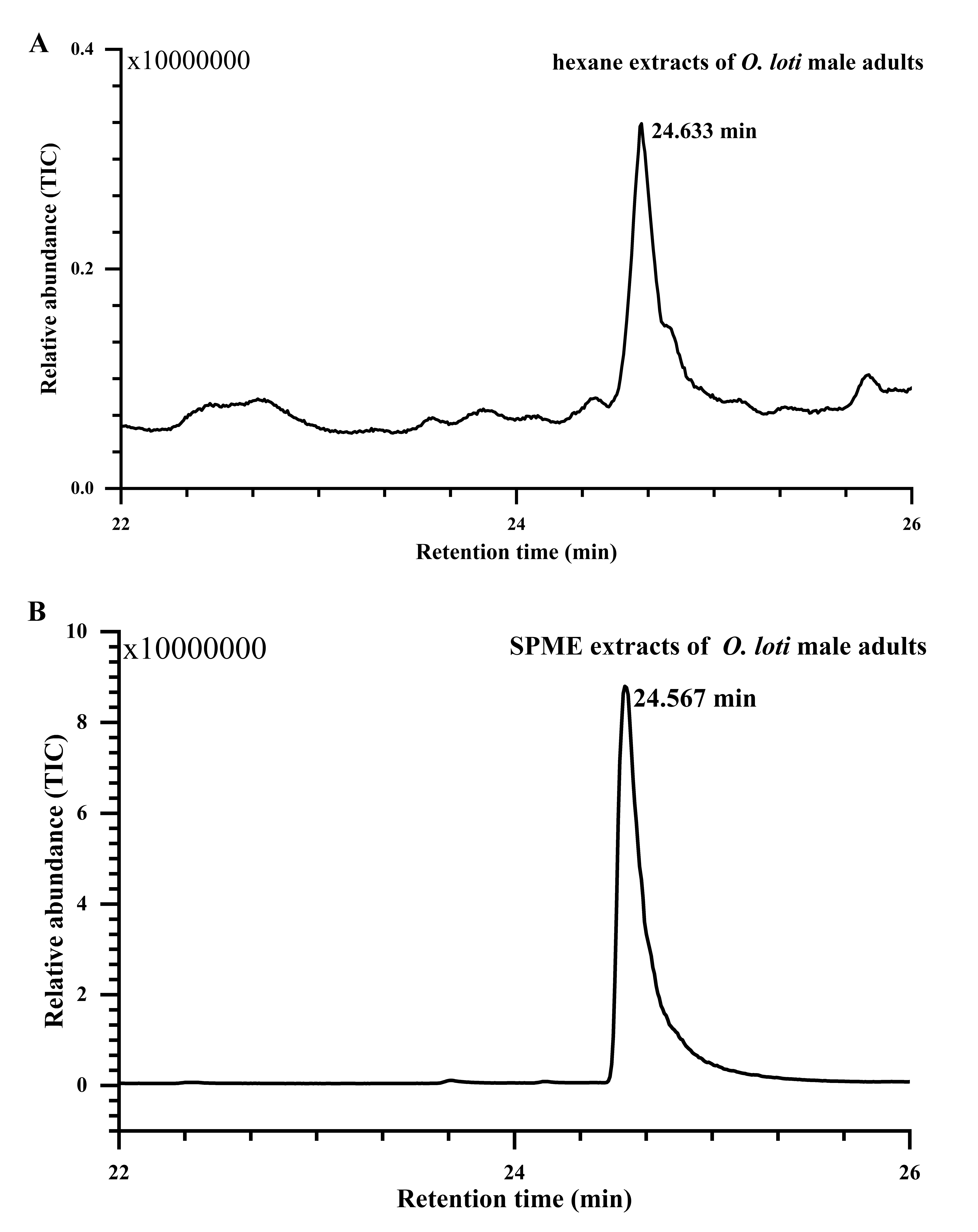

Supplement: Supplementary file 1 — Supplementary Material 1 [file 10886_2024_1532_MOESM1_ESM.tiff]
